# Risk Prediction After Myocardial Infarction by Cyclic Variation of Heart Rate, a Surrogate of Sleep-Disordered Breathing Assessed From Holter ECGs

**DOI:** 10.3389/fphys.2019.01570

**Published:** 2020-01-15

**Authors:** Xu Cao, Alexander Müller, Ralf J. Dirschinger, Michael Dommasch, Alexander Steger, Petra Barthel, Karl-Ludwig Laugwitz, Georg Schmidt, Daniel Sinnecker

**Affiliations:** ^1^Klinik und Poliklinik für Innere Medizin I, University Hospital Klinikum Rechts der Isar, Technical University of Munich, Munich, Germany; ^2^DZHK (German Centre for Cardiovascular Research), Partner Site Munich Heart Alliance, Munich, Germany

**Keywords:** myocardial infarction, sleep-disordered breathing, cyclic variation of heart rate, risk stratification, heart rate variability

## Abstract

**Aims:**

Sleep-disordered breathing (SDB) is common among cardiac patients, but its role as an independent risk predictor after myocardial infarction (MI) is unclear. SDB causes cyclic variation of heart rate (CVHR). The aim of this study was to score Holter ECGs of a large cohort of MI survivors for SDB-related CVHR to investigate its value for mortality prediction.

**Methods:**

A total of 1590 survivors of acute MI in sinus rhythm were prospectively enrolled and followed for 5-year all-cause mortality. Heart rate (HR) tachograms were generated from nocturnal (00:00–06.00 am) segments of Holter ECGs, and the minutes with CVHR were quantified by a previously developed algorithm. According to a pre-specified cutpoint, SDB was assumed if CVHR was present during ≥72 min.

**Results:**

Seventy-seven patients (4.8%) had flat HR tachograms which prohibited analysis for SDB. Of the remaining 1513 patients, 584 (38.6%) were classified as having SDB. Mortality rates in groups stratified according to ECG-derived SDB did not differ significantly. Taken as a continuous variable, low CVHR duration was associated with increased mortality.

The mortality of patients with flat HR tachograms was significantly increased, even after adjustment for age, sex, LVEF, GRACE score and diabetes mellitus. Mortality prediction by a flat HR tachogram was also independent of heart rate variability (HRV), heart rate turbulence (HRT), and deceleration capacity (DC).

**Conclusion:**

In Holter ECG recordings of survivors of acute MI, signs suggestive of SDB were frequently present, but not associated with mortality. A flat nocturnal HR tachogram was a strong, independent predictor of 5-year all-cause mortality.

## Introduction

Patients who have survived the acute phase of an acute myocardial infarction (MI) are at increased risk of subsequent mortality within the next years, which may be due to re-infraction, arrhythmias, or progressive heart failure, but also related to co-morbidities. Identification of high-risk MI survivors is a crucial part of subsequent care and secondary prophylactic therapy. Current approaches to assess the mortality risk of these patients include clinical scores (e.g., GRACE risk score) (Eagle et al., 2004) screening for co-morbidities (e.g., diabetes mellitus, renal impairment), measurement of the left-ventricular ejection fraction (LVEF), (Dagres and Hindricks, 2013) or evaluation for parameters of cardiac electric instability or cardiac autonomic dysfunction [e.g., heart rate variability (HRV), (Task Force of the European Society of Cardiology and the North American Society of Pacing and Electrophysiology, 1996) heart rate turbulence (HRT), (Schmidt et al., 1999) deceleration capacity of heart rate (DC), (Bauer et al., 2006) or severe autonomic failure (SAF, a combination of abnormal HRT and abnormal DC) (Bauer et al., 2009)].

Recently, several parameters related to respiration such as the respiratory rate (Barthel et al., 2013) [which can be also measured from Holter ECG recordings as the nocturnal respiratory rate (Dommasch et al., 2014; Sinnecker et al., 2014)] or respiratory sinus arrhythmia (Sinnecker et al., 2016) have been demonstrated to be strong predictors of the mortality risk of MI survivors.

Sleep-disordered breathing (SDB), which is an important risk factor for cardiovascular events such as MI, (Marin et al., 2016) is frequently found in MI survivors (Porto et al., 2017). The question whether the presence of SDB in survivors of acute MI bears implications for the patients’ prognosis has been so far investigated in some small clinical studies, with conflicting results (Ludka et al., 2017; Shah et al., 2017). It has been recognized that SDB is accompanied by a typical pattern of heart rate decelerations (during apnea episodes) followed by accelerations (during arousals) which has been termed cyclic variation of heart rate (CVHR), (Guilleminault et al., 1984) and various algorithms have been proposed to assess the likelihood of SDB from standard Holter ECG recordings (Mendonca et al., 2018). Proprietary algorithms to perform this kind of analysis have been also implemented into commercial Holter equipment. Interestingly, a simple method to manually score RR interval tachograms generated from ECG recordings obtained during polysomnography for the presence of CVHR (Stein et al., 2003) was able to predict the presence or absence of SDB (as assessed by standard polysomnography) with a positive and negative predictive accuracy of 86 and 100%, respectively (Stein et al., 2003).

The aim of this work was to assess whether CVHR, assessed with the above mentioned method (Stein et al., 2003) from nocturnal Holter ECG recordings in a large cohort of post-infarction patients, bears prognostic information, and, if so, whether the previously described association of an increased nocturnal respiratory rate with an increased risk of non-sudden cardiac death (Dommasch et al., 2014) is related to SDB.

## Materials and Methods

### Study Cohort

The study is a retrospective analysis of a previously described prospective cohort study (Bauer et al., 2009; Dommasch et al., 2014; Sinnecker et al., 2014) aimed at assessing risk predictors of subsequent mortality in survivors of the acute phase of MI. Patients admitted with acute MI at one of two centers (German Heart Centre and Klinikum rechts der Isar, both in Munich, Germany) were enrolled between May 2000 and March 2005. Inclusion criteria were (i) age ≤80 years, (ii) sinus rhythm at admission, (iii) MI within 4 weeks before enrollment, (iv) survival until hospital discharge, and (v) no indication for ICD implantation for secondary prophylaxis at the time of enrolment. Diagnosis of acute MI required two or more findings of: (i) chest pain for ≥20 min, (ii) creatine kinase more than twice the upper limit of normal, and (iii) and ST-segment elevation ≥0.1 mV in two or more limb leads or ≥0.2 mV in two or more contiguous precordial leads on admission. All study subjects underwent Holter ECG recordings during the first days after MI. Patients were followed-up for all-cause mortality for 5 years. The study was approved by the local ethics committee, and informed consent was obtained from all participants.

### ECG Analysis

Holter ECG recordings (Oxford Excel Holter system, Oxford instruments; Pathfinder 700, Reynolds Medical; and Mortara Holter system, Mortara Instrument) were performed at a median of 5 days (IQR:4–7 days) after MI, with a median recording period of 21 h (IQR 19–23 h). The recordings were first automatically analyzed to annotate beats. In a second step, each recording was reviewed by a physician who corrected the annotations where necessary and removed segments that were not analyzable due to noise or technical problems.

### Assessment of Cyclic Variation of Heart Rate Indicating SDB

Sleep-disordered breathing was assessed from the Holter ECGs using a previously developed method (Stein et al., 2003). Care was taken to faithfully follow the published method, which is based on manual annotation and scoring of tachograms generated from the Holter ECGs. Due to the large number of ECGs in our study, we did not print out the tachograms on paper and marked CVHR episodes with a pen as originally suggested (Stein et al., 2003), but rather inspected the tachograms on a computer screen and annotated the CVHR episodes using a mouse. To this end, a custom-written analysis software was used that plotted the instantaneous heart rate (i.e., the inverse of normal-to-normal intervals) to a computer screen and allowed manual annotation of CVHR episodes, which were stored in a database for further analysis. We restricted the analysis to a nocturnal 6-h segment (00:00–06.00 am) of the recordings, assuming that most of the patients would be asleep during this time. The analysis was performed by a physician with experience in biosignal analysis.

**FIGURE 1:**
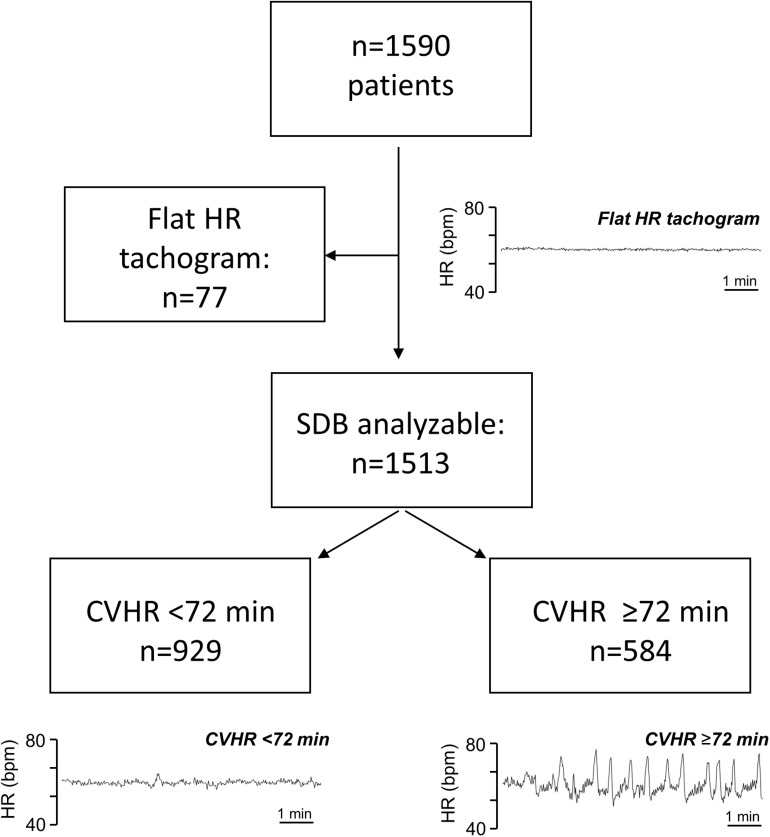
Patient flow chart. Typical Examples of nocturnal HR tachogram segments from patients with a flat tachogram, with CVHR <72 min, and with CVHR ≥72 min are shown as indicated. HR, heart rate; bpm, beats per minute; SDB, sleep-disordered breathing; CVHR, cyclic variation of heart rate.

As previously described (Stein et al., 2003), some patients had a flat HR tachogram during the analyzed nocturnal segment, which was defined as a tachogram having (1) no changes of the instantaneous heart rate exceeding ≥5 beats/min, and (2) no visible respiratory sinus arrhythmia (Figure 1). In patients with a flat tachogram, it is not possible to obtain information on the presence of SDB from ECG recordings (Stein et al., 2003). In all recordings not exhibiting any CVHR episodes (see below), the presence or absence of a flat nocturnal tachogram according to the above definition was independently judged by two physicians, and a consensus regarding the presence or absence of a flat nocturnal tachogram was reached in each of these cases. All persons involved in the analysis of the biosignals were blinded to the outcome of the patients.

Recordings with a non-flat tachogram were evaluated for episodes of CVHR, which were defined as previously described (Stein et al., 2003) as ECG segments containing ≥3 successive HR increases (arousals) of at least 6 bpm and at least 10 s duration, with ≤2 min between two successive arousals. The total duration of the CVHR episodes during a recording was calculated based on the manual annotation of CVHR episodes. According to the pre-specified cutpoint, SDB was assumed if CVHR episodes were present in ≥20% of the recording, (Stein et al., 2003) which corresponds to ≥72 min of the nocturnal 6-h segment.

### Mean Nocturnal Respiratory Rate

Nocturnal respiratory rate was automatically derived from Holter recordings by a previously described algorithm (Dommasch et al., 2014; Sinnecker et al., 2014) analyzing beat-to-beat changes in QRS amplitudes, QRS vectors, and RR intervals.

### Heart Rate Variability and Severe Autonomic Failure

Parameters assessing HRV (time-domain and frequency-domain parameters, HRT, DC) were determined from the Holter recordings using standard methods (Task Force of the European Society of Cardiology and the North American Society of Pacing and Electrophysiology, 1996; Sassi et al., 2015). According to a previous report, (Bauer et al., 2009) SAF was defined as the combination of both abnormal HRT (slope ≤2.5 ms/RR and onset ≥0%) and abnormal cardiac DC (≤4.5 ms).

### Left-Ventricular Ejection Fraction

Left-ventricular ejection fraction was measured within 2 weeks after MI by angiography or biplane echocardiography (Sonos 5500, Hewlett Packard) based on Simpson’s method.

### GRACE Score

The clinical GRACE score to predict the long-term mortality risk was calculated based on age, history of past heart failure and MI, serum creatinine and the cardiac biomarker status at admission, pulse and systolic blood pressure at admission, ST segment deviation and in-hospital percutaneous coronary intervention as described previously (Eagle et al., 2004).

### Endpoint of the Study

The primary endpoint was all-cause mortality within 5 years after index MI. Follow-up was conducted with clinical appointments every 6 months. Participants who missed these appointments were contacted by letter, telephone, or through their General Practitioner. If necessary, the local population registry was contacted to obtain the new address in case participants changed residence, or to confirm death in case participants deceased.

### Statistical Analysis

Continuous variables are presented as median and inter-quartile range (IQR), and categorical data are presented as frequency and percentage.

The linear correlation of two sets of data points was assessed by Pearson correlation coefficient. Survival curves were generated using the Kaplan–Meier method and compared with the log-rank test. Univariable or multivariable Cox analysis was performed to calculate hazard ratios (with 95% confidence intervals) for 5-year all-cause mortality.

R 3.0.1 (R Foundation for Statistical Computing, Vienna, Austria) was used for all statistical calculations. Differences were considered statistically significant if *p* < 0.05.

## Results

A total of 1590 patients hospitalized for acute MI met the inclusion criteria and were included in the analysis. Out of these, 1513 patients had a “non-flat tachogram,” i.e., it was possible to score the heart rate tachogram for SDB-related CVHR (Figure 1). Of these patients, 584 had CVHR episodes in ≥72 min of the analyzed 6-h segment, indicating presence of SDB, while 929 were classified as not having SDB. The remaining 77 patients showed a “flat tachogram” (see Figure 1).

The clinical and demographic characteristics of patients with “flat” and “non-flat” HR tachograms are shown in Table 1. Patients with a “flat tachogram” were older, more often female, had a higher GRACE score, a lower LVEF, and a higher prevalence of diabetes mellitus. The first part of the section “Results” will focus on the patients with a “non-flat tachogram” to assess the prognostic implications of Holter-derived screening for SDB. The second part will investigate the prognostic implications of a “flat tachogram.”

**TABLE 1:** Baseline characteristics.

**Variable**	**All patients (*n* = 1590)**	**“Non-flat tachogram” (*n* = 1513)**	**“Flat tachogram” (*n* = 77)**	***p*-Value (“flat” vs.“non-flat” tachogram)**	**“Non-flat tachogram”**		***p*-Value (CVHR <72 min vs. CVHR ≥72 min)**
						
					**CVHR <72 min (*n* = 929)**	**CVHR ≥72 min (*n* = 584)**	
Age (median [IQR])	59.2 [51.6–66.8]	58.6 [51.2–66.3]	67.2 [59.7–73.3]	<0.001	60.3 [54.0–67.7]	55.9 [48.5–64.1]	<0.001
Females, *n* (%)	327 (20.6)	302 (20.0)	25 (32.5)	0.012	216 (23.3)	86 (14.7)	<0.001
Acute intervention, n (%)				0.051			0.47
PCI	1451 (91.3)	1384 (91.5)	67 (78.0)		840 (90.4)	544 (93.2)	
CABG	33 (2.1)	30 (2.0)	3 (3.9)		22 (2.4)	8 (1.4)	
Thrombolysis	54 (3.4)	53 (3.5)	1 (1.3)		37 (4.0)	16 (2.7)	
None	52 (3.3)	46 (3.0)	6 (7.8)		30 (3.2)	16 (2.7)	
Diabetes mellitus, *n* (%)	270 (17)	249 (16.2)	32 (41.6)	<0.001	158 (17.0)	80 (13.7)	0.099
LVEF (median [IQR])	55 [45–63]	55 [45–63]	48 [39–57]	<0.001	55 [45–63]	56 [46–63]	0.836
GRACE Score [mean (sd)]	96 (80–113)	96 (79–112)	114 (103–125)	<0.001	99 (82–113)	90 (75–108)	<0.001
CK_*max*_, U/l (median [IQR])	1140 [569–2478]	1140 [569–2478]	1082 [566–2420]	0.856	1110 [560–2429]	1200 [586–2518]	0.33
Creatinine, mg/dl (median [IQR])	1.1 [0.9–1.3]	1.1 [0.9–1.3]	1.2 [1.0–1.5]	0.002	1.1 [1.0–1.3]	1.1 [0.9–1.2]	0.019

*IQR, inter-quartile range; PCI, percutaneous coronary intervention; CABG, coronary artery bypass grafting; LVEF, left-ventricular ejection fraction; GRACE, Global Registry of Acute Coronary Events; CKmax, maximal creatine kinase plasma concentration.*

### Patients With a “Non-flat Tachogram”

**FIGURE 2:**
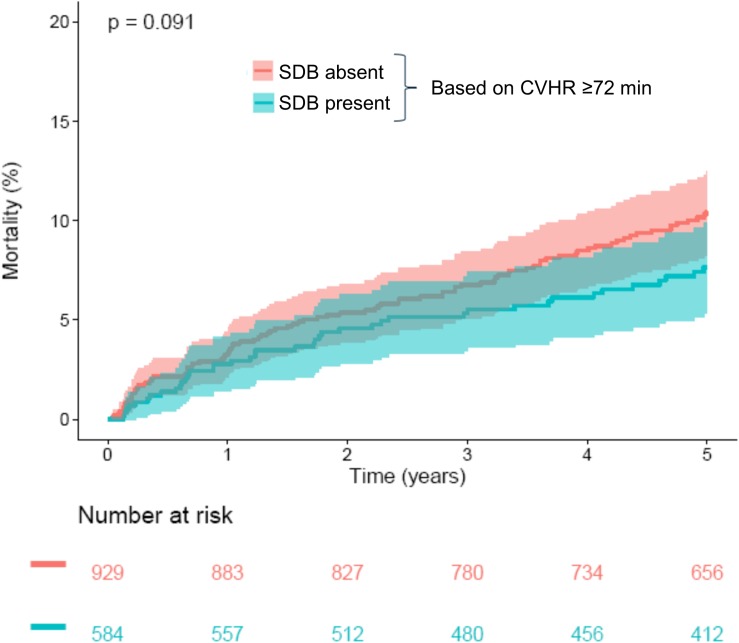
Survival in patients stratified according to CVHR. Kaplan–Meier curves along with 95% confidence intervals are shown for patients with CVHR <72 min (“SDB absent”) and ≥72 min (“SDB present”). The numbers of patients at risk in the respective groups are shown below the graph. CVHR, cyclic variation of heart rate; SDB, sleep-disordered breathing.

CVHR ≥72 min, indicating presence of SDB, was found in 584/1513 patients (38.6%). Compared to the group with <72 min of CVHR, indicating absence of SDB, these patients were younger, more often male, and had a significantly lower GRACE score, whereas no significant differences were present regarding other risk factors (Table 1). Five-year all-cause mortality did not differ significantly between these two groups (Figure 2).

**FIGURE 3:**
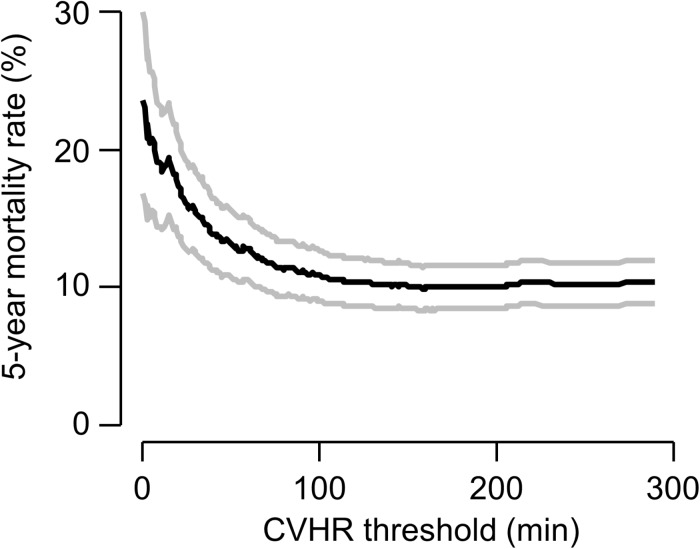
Continuous association of CVHR with mortality. The 5-year mortality rate (black curve) estimated by the Kaplan–Meier method in patients with a non-flat tachogram with a CVHR duration less than or equal to a certain threshold are plotted as a function of this threshold. Gray curves show 95% confidence limits of the mortality estimate.

As an exploratory analysis, we investigated the effect of the overall duration of CVHR episodes, treated as a continuous variable, on 5-year mortality (Figure 3). Interestingly, very short CVHR durations, markedly below the threshold of 72 min that indicates absence of sleep apnea, were associated with substantially increased mortality rates (see Figure 3).

**FIGURE 4:**
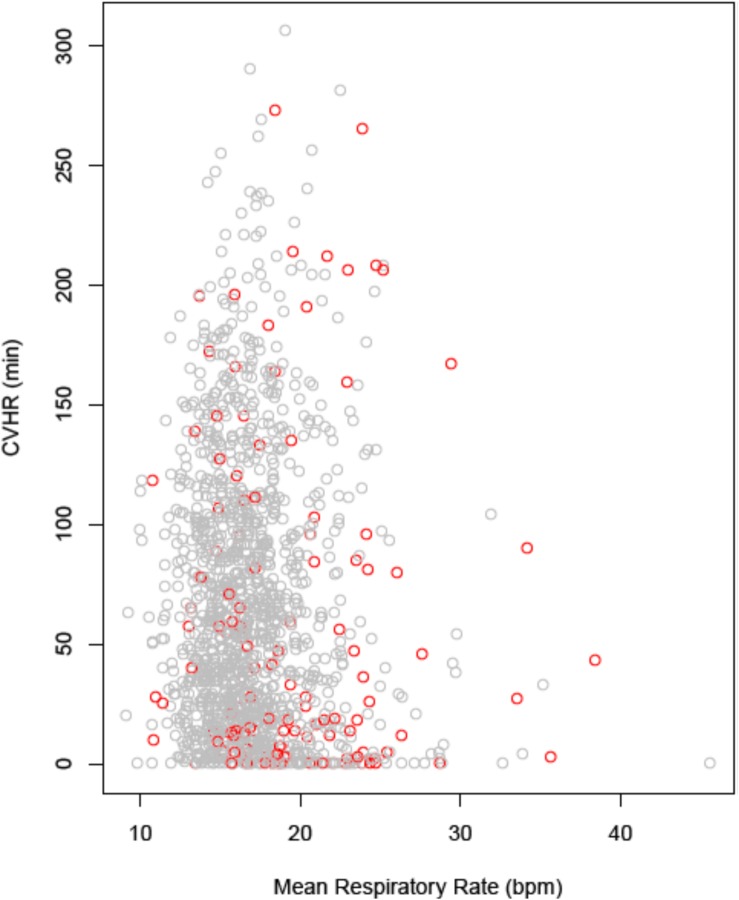
Correlation between nocturnal CVHR and respiratory rate. CVHR is expressed as the number of minutes with CVHR within the nocturnal 6-h segment, respiratory rate as the mean value over the same segment. Data points from patients who were alive at 5 years are printed in gray, data points from deceased patients in red. CVHR, cyclic variation of heart rate; bpm, breaths per minute.

There was no substantial correlation between continuous CVHR duration and the nocturnal respiratory rate (NRR) determined from the identical segment of the ECG recording (Figure 4; correlation coefficient −0.068). In multivariable Cox regression, NRR (hazard ratio 3.1; *p* = < 0.0001), but not CVHR (hazard ratio 0.99; *p* = 0.31) was a significant mortality predictor in our cohort.

### Patients With a “Flat Tachogram”

**FIGURE 5:**
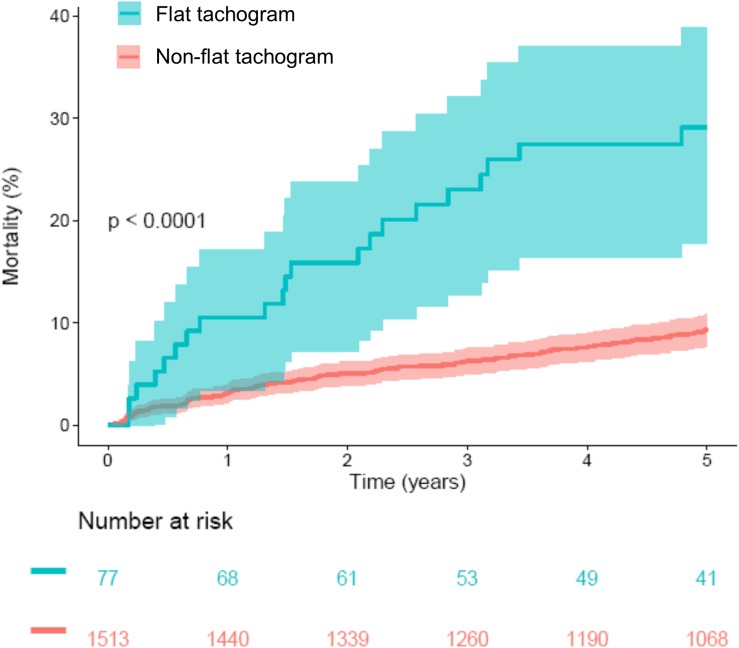
Survival in patients stratified according to presence or absence of a flat heart rate tachogram. Kaplan–Meier curves along with 95% confidence intervals are shown. The numbers of patients at risk in the respective groups are shown below the graph. CVHR, cyclic variation of heart rate; SDB, sleep-disordered breathing.

A comparison of the mortality rates in patients with a “flat tachogram” and in the remaining patients revealed that a “flat tachogram” identifies a high-risk population with a substantially increased 5-year mortality rate [29.1% (95% CI 17.7–38.9) vs. 9.4% (95% CI 7.8–10.9); *p* < 0.0001; Figure 5]. This may be partly explained by the above-mentioned differences in baseline risk factors. However, in multivariable Cox analysis considering also age, sex, LVEF, GRACE score, and diabetes mellitus, a “flat tachogram” was independently associated with mortality with a hazard ratio of 1.7 (*p* = 0.022) (Table 2).

**TABLE 2:** Multivariable Cox analysis.

**Variable**	**Hazard ratio (95% CI)**	***p*-Value**
Flat HR tachogram	1.73 (1.08−2.78)	0.022
Age (per year)	1.06 (1.03−1.08)	< 0.0001
Female sex	0.98 (0.67−1.44)	0.92
LVEF (per %)	0.95 (0.93−0.96)	< 0.0001
GRACE score (per point)	1.004 (0.99−1.01)	0.47
Diabetes mellitus	1.66 (1.17−2.35)	0.005

*HR, heart rate; LVEF, left-ventricular ejection fraction; GRACE, Global Registry of Acute Coronary Events.*

**FIGURE 6:**
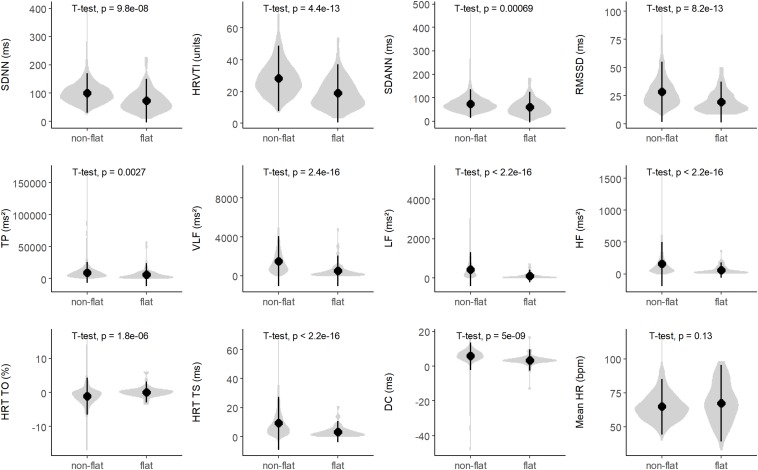
Distribution of heart rate variability (HRV) parameters in patients with non-flat and flat nocturnal tachograms. The gray violin plots illustrate kernel probability density, while the black circles and vertical lines indicate means and 95% confidence intervals, respectively. SDNN, standard deviation of all normal-to-normal intervals; HRVTI, HRV triangular index; SDANN, standard deviation of the averages of NN intervals in 5 min segments; RMSSD, the square root of the mean of the sum of the squares of differences between adjacent NN intervals; TP, total power; VLF, very low frequency; LF, low frequency; HF, high frequency; HRT, heart rate turbulence; TO, turbulence onset; TS, turbulence slope; DC, deceleration capacity; HR, heart rate.

A “flat tachogram” can be considered indicative of a markedly reduced HRV. It is well-known that reduced HRV signifies an increased risk in cardiac patients (Task Force of the European Society of Cardiology and the North American Society of Pacing and Electrophysiology, 1996; Sassi et al., 2015). To investigate whether the presence of a “flat tachogram” simply signifies reduced HRV that could be captured also by assessing classical HRV parameters (SDNN, HRVTI, SDANN, RMSSD, TP, VLF, LF, HF; see legend to Figure 6 for an explanation of the abbreviations), we first investigated the distribution of these parameters in patients with “flat” and “non-flat” tachograms (Figure 6). Indeed, the mean values of all these HRV parameters were significantly lower in the patients with a “flat tachogram.” However, the distributions of all these HRV parameters showed a high degree of overlap between patients with “flat” and “non-flat” nocturnal tachograms (see Figure 6). In univariable Cox models, each of the parameters shown in Figure 6 was a significant predictor of 5-year mortality. In pairwise multivariable Cox models considering each of these parameters together with the presence of a “flat nocturnal tachogram,” the flat tachogram always remained an independent mortality predictor (data not shown), corroborating that its presence conveys prognostic information not captured by classical HRV parameters. All heart rate variability parameters shown in Figure 6, together with mortality status at 5 years, flat nocturnal tachogram status, and the number of minutes with CVHR during the nocturnal segment for all patients are given in Supplementary Table S1.

**FIGURE 7:**
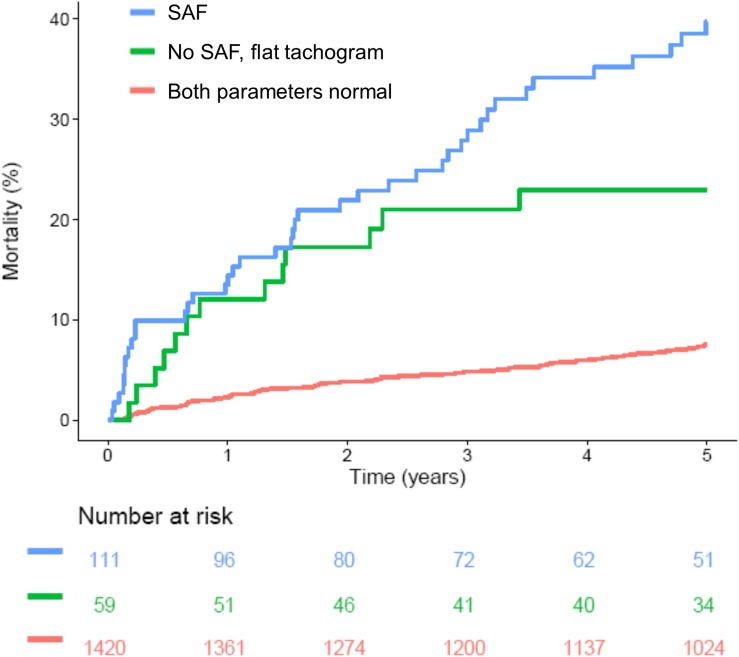
Survival in patients stratified according to presence of a flat heart rate tachogram and severe autonomic failure (SAF). Kaplan–Meier curves are shown for patients without a flat tachogram and SAF (“Both parameters normal”), for patients with SAF regardless of flat tachogram status (“SAF”) and for the subgroup of patients without SAF, but with a flat nocturnal HR tachogram (“No SAF, flat tachogram”). The numbers of patients at risk in the respective groups are shown below the graph.

More recently introduced parameters based on the variation of RR intervals such as HRT (Schmidt et al., 1999) or DC (Bauer et al., 2006) provide much stronger risk stratification information than classical HRV parameters (the distribution of these parameters in patients with “non-flat” and “flat” nocturnal tachograms is also shown in Figure 6). However, in our cohort, presence of a “flat tachogram” even provided additional prognostic value when added to “severe autonomic failure” (SAF), a parameter combining HRT and DC (Bauer et al., 2009): of the 1590 patients in our cohort, 111 had SAF, and 77 had a “flat tachogram.” The overlap between these two groups was small; only 18 patients had both, a “flat tachogram” and SAF. Presence of SAF identified a high-risk group of 111 patients who had a 5-year mortality risk of 40% (Figure 7). However, the presence of a “flat tachogram” in patients without SAF identified another high-risk group of 59 patients with an almost similarly high mortality rate (see Figure 7). In a multivariable Cox model considering also LVEF and GRACE score, SAF and a “flat tachogram” were independent risk predictors with hazard ratios of 2.9 (*p* < 0.001) and 1.9 (*p* = 0.013), respectively, indicating that the presence of a “flat tachogram” provides additional prognostic information in survivors of acute MI.

## Discussion

In this study, we applied a previously developed algorithm to screen nocturnal segments from Holter recordings of a large cohort of survivors of acute MI for SDB based on CVHR. The primary result of our study was that a nocturnal ECG pattern indicative of SDB did not have prognostic implications regarding 5-year mortality rate in this patient cohort (see Figure 2).

Of those patients in whom the algorithm to detect SDB from the ECG could be applied, 38.6% had a nocturnal ECG pattern indicative of SDB in this cohort of post-infarction patients. This fits well with numerous evidence referring to a high prevalence of SDB among cardiac patients [e.g., 30–50% of patients with coronary heart disease (Andreas et al., 1996; Hayano et al., 2017)] and underscores the importance of this disease entity in post-infarction patients.

In some patients (4.8% in our cohort), a “flat” heart rate tachogram does not allow an evaluation for signs of SDB. Considering that reduced HRV, as a sign of cardiac autonomic dysfunction, is a known predictor of adverse outcome, (Task Force of the European Society of Cardiology and the North American Society of Pacing and Electrophysiology, 1996) it might be speculated that the “flat tachogram” is also indicative of cardiac autonomic dysfunction and poor prognosis. Indeed, patients with a “flat tachogram” were characterized by a higher burden of cardiovascular risk factors at baseline (see Table 1). In a multivariable logistic regression analysis considering all baseline parameters shown in Table 1, only advanced age, presence of diabetes mellitus and reduced LVEF were significantly associated with the presence of a “flat” nocturnal tachogram (data not shown).

Interestingly, however, this “flat tachogram” was a strong risk predictor even after adjustment for baseline risk factors (see Figure 7 and Table 2), providing prognostic information independent from both, “classical” HRV parameters and more recently introduced parameters such as HRT and DC. This unexpected result warrants further investigation. We hypothesize that SDB is a strong stimulus for the sympatho-vagal regulatory circuits influencing the heart rate, and that some of our patients with SDB cannot react to this stimulus (i.e., do not develop CVHR) because of severe impairment of the cardiac autonomic system. The existence of such a mechanism has been demonstrated in a cohort of coronary artery disease patients with preserved (LVEF >50%) and reduced (LVEF ≤35%) left-ventricular function who underwent sleep studies using a Holter ECG and pulse oximetry (Keyl et al., 2017): In patients with preserved LVEF, sleep-apnea-related nocturnal periods of cyclic variations in the oxygen saturation were accompanied by significantly increased HRV and a shift of the sympatho-vagal balance toward sympathetic predominance, whereas in patients with reduced LVEF, this autonomic response to cyclic oxygen desaturations was blunted. Our hypothesis is also in line with a recent report in which the amplitude of the HR fluctuations related to CVHR was investigated by phase-rectified signal averaging (Hayano et al., 2017). This amplitude, rather than the frequency of CVHR episodes, was associated with mortality in different patient cohorts with MI, chronic heart failure, and end-stage renal disease, with a lower amplitude of CVHR indicating an increased mortality risk (Hayano et al., 2017). Interestingly, also in this study, 3.6% of the patients did not have enough CVHR episodes to calculate CVHR amplitude (similar to our 4.8% “flat tachogram” patients, even though the definition of this patient group was slightly different). The mortality risk of this subgroup, however, was not reported.

An exploratory analysis of our data was also consistent with the hypothesis that, at least in a substantial fraction of patients, reduced CVHR might signify cardiac autonomic impairment rather than absence of SDB: For the above-reported results, we used the pre-defined cutoff of CVHR in ≥72 min of the analyzed time segment to assume presence of SDB (Stein et al., 2003). However, in our data, there was a continuous association of CVHR duration and increased mortality risk (see Figure 3), and the optimal cutpoint with regard to risk stratification would be rather ≤19 min within the 6-h segment. Using this cutpoint, a significant association between CVHR and 5-year mortality was present (data not shown). Notably, a lower CVHR duration was associated with increased risk, i.e., the high-risk group consisted of those patients with CVHR in 19 min or less of the analyzed time segment. We hypothesize that cardiac autonomic impairment, by blunting the heart rate response to CVHR, reduces the number of apnea episodes detectable by CVHR.

Presence of a “flat nocturnal heart rate tachogram” can be easily assessed from standard Holter ECG recordings. It would be interesting to investigate whether its presence provides similar independent prognostic information in other cohorts, or whether the prognostic implications of other ECG-derived parameters profit from only investigating them during a nocturnal time segment.

However, since no polysomnograms were performed in our patients, we do not know how many of the patients actually had SDB. Thus, the study does not allow to make any statement on whether the presence of SDB (as opposed to “a nocturnal ECG pattern suggestive of SDB”) is an independent risk factor in survivors of acute MI. It would be very informative to obtain polysomnogram data from a similar cohort and subsequently follow the patients for endpoints such as mortality, which could answer this question.

Nonetheless, our results suggest that screening for SDB by means of ECG analysis does not provide any meaningful prognostic information in addition to the “classical” risk factors. The fact that the patients with a “flat tachogram” – in whom ECG-based screening for SDB is not possible – are a patient population with an extraordinarily high 5-year mortality risk should raise caution regarding the use of ECG-based SDB detection tools in post-MI patients. If they are used, one should consider performing additional tests, e.g., portable cardiorespiratory polygraphy or polysomnography, in patients with a “flat tachogram” in the Holter ECG to avoid missing possibly important information in a particularly vulnerable patient group. However, even in patients who have a “non-flat” tachogram, one cannot decide, based on the ECG recordings alone, whether a very infrequent occurrence of CVHR solely signifies absence of SDB, or rather severe cardiac autonomic dysfunction.

In our previous study, which revealed that post-MI patients with an increased nocturnal respiratory rate are at increased risk of non-sudden cardiac death (Dommasch et al., 2014; Sinnecker et al., 2014) we could not exclude the possibility that this association is (at least partly) mediated by SDB. Our present study indicates that there is no correlation between SDB and the nocturnal respiratory rate.

### Limitations

All the patients enrolled in our study were in sinus rhythm. Thus, we cannot make any statement on the utility of the algorithm in patients with atrial fibrillation. Similarly, we only investigated patients with a recent MI, and it may not be valid to extrapolate our findings to patients with other cardiac conditions.

Since we did not directly measure respiration in our patients, we cannot exclude that SDB that escaped detection by the screening algorithm (possibly in the patients with a “flat tachogram”) bears prognostic information after MI. Similarly, since our algorithm does not allow to discriminate between patients with obstructive (OSA) and central (CSA) sleep apnea, we cannot make any statement on differential prognostic implications of these two forms of SDB. It is also possible that part of the observed CVHR arises from sleep fragmentation unrelated to sleep apnea, which is common among hospitalized patients (Macfarlane et al., 2019).

The high-risk group defined by a flat nocturnal tachogram is small (only 4.8% of the whole cohort). This may limit the power of statistical analyses comparing this group to the remaining patients.

## Conclusion

In survivors of acute MI, Holter recordings suggesting SDB are frequently seen. In our cohort, this specific pattern was not associated with an altered mortality rate. A nocturnal “flat tachogram” — indicating impaired autonomic control of heart rate, and making Holter-based screening for SDB impossible — was a strong and independent mortality predictor after MI. Thus, Holter-based detection methods for sleep apnea should be used with caution in cardiac patients.

## Data Availability Statement

The datasets generated for this study are available on request to the corresponding author.

## Ethics Statement

The studies involving human participants were reviewed and approved by the Ethics Committee of the Klinikum rechts der Isar der Technischen Universität München. The patients/participants provided their written informed consent to participate in this study.

## Author Contributions

DS planned and coordinated the study and performed final data analysis. XC, AM, RD, AS, and MD analyzed the data. GS and PB planned, performed, and supervised the prospective clinical study on which this subanalysis is based. GS and K-LL provided administrative oversight and support. DS and XC wrote the first version of the manuscript, with input from all authors.

## Conflict of Interest

The authors declare that the research was conducted in the absence of any commercial or financial relationships that could be construed as a potential conflict of interest.
